# Health professionals’ communication and Italian pregnant women’s attitudes to the Covid-19 vaccine

**DOI:** 10.1093/eurpub/ckac130.244

**Published:** 2022-10-25

**Authors:** C Fiammenghi, NA Mbaye, D Pelleri, U Gelatti, L Covolo

**Affiliations:** 1 Unit of Hygiene, Epidemiology, Public Health, University of Brescia, Brescia, Italy; 2 Degree Course in Health Assistance, University of Brescia, Brescia, Italy

## Abstract

**Background:**

Pregnancy is a risk condition for hospitalization and severe illness from Covid-19, with an increased risk of maternal mortality and serious neonatal complications. The study examines Italian pregnant women's attitudes about the Covid-19 vaccine, the role of healthcare professionals’ (HP) communication, the reasons and potential predictors for non-adherence to vaccination.

**Methods:**

An online survey was developed by LimeSurvey software and spread through social media between August 2021 and January 2022 to pregnant women of age living in Italy. Participants were asked to indicate their sources of information and to rate the support received from their HP; their health literacy (HL) was assessed using the HLS-EU-Q6 tool. Multivariate linear regression analysis was performed. Open-ended questions were analysed using MaxQDA 2022.

**Results:**

1594 total survey responses were obtained (median age 31.5±4.94); 48% of the participants had a university degree. Only 17% of women had sufficient HL. Most (52%) of them refused to be vaccinated against Covid-19 while pregnant, 27% were unsure and 26% disagreed about the safety of the vaccine during pregnancy. Most of them did not deem the information received by HP complete (56%), clear (52%), and reliable (46%); 49% of them did not feel supported in their decision to vaccinate. This variable was the main predictor of vaccine hesitancy in addition to concern about vaccine safety in the multivariate model. Among women who felt unsatisfied 57% had an inadequate HL compared to 40% of those who had sufficient HL (p<.0001). The analysis of the open-ended questions also revealed a pervasive feeling of uncertainty.

**Conclusions:**

The study highlights how the lack of adequate communication and support by HP had a strong impact in the adherence to Covid-19 vaccination among pregnant women.

**Key messages:**

- The lack of an effective communication by healthcare professionals contributed to the feeling of uncertainty and concern of pregnant women about Covid-19 vaccination.
- There is a need to put effort on training for HP to improve their communication skills to support pregnant women’s health decisions and improve their literacy in such a delicate phase of their life.

